# Types of reproductive disorders in underweight and overweight young females and correlations of respective hormonal changes with BMI

**Published:** 2015-03

**Authors:** Nutsa Aladashvili-Chikvaidze, Jenara Kristesashvili, Manana Gegechkori

**Affiliations:** 1 *Department of Obstetrics, Gynecology and Reproductology, Faculty of Clinical and Translational Medicine, I. Javakhishvili Tbilisi State University, Tbilisi, Georgia.*; 2 *Department of Obstetrics, Gynecology and Reproductology, I. Javakhishvili Tbilisi State University, Center of Reproductive Medicine “Universe”, Tbilisi, Georgia.*; 3 *I. Zhordania Institute of Human Reproduction, Tbilisi, Georgia.*

**Keywords:** *Body mass index*, *Body weight changes*, *Childhood obesity*, *Thinness*, *Reproductive health*

## Abstract

**Background:**

Higher risks of reproductive problems have been found in underweight and overweight women with rapid weight gain or loss but evidence is inconsistent especially in relation to the effect of age of body weight changes.

**Objective:**

The aim of our study was to detect the peculiarities of menstrual function, prevalence of different types of reproductive disorders and correlations of respective hormonal changes with body mass index (BMI) in young female patients with thinness or obesity since childhood.

**Materials and Methods:**

In this prospective cross-sectional study 48 underweight and 55 overweight/obese young women with different reproductive problems underwent complete clinical and hormonal analyses. All 103 patients had weight problems since childhood.

**Results:**

Polycystic ovarian syndrome and metabolic syndrome was the most frequent in overweight and obese women, whilst non-classical congenital adrenal hyperplasia and ovarian dysfunction prevailed in underweight women (p˂0.001). No difference was determined according to the age of menarche (p=0.885) and types of menstrual disturbances (p=0.34) between the study groups. Hypogonadotropic hypogonadism was not found in young women who were lean since childhood. Follicle-stimulating hormone (FSH) (p=0.013) and sex hormone binging globulin (SHBG) (p˂0.001) levels were higher in women with low BMI, whilst free testosterone (FT) (p=0.019) and total testosterone (TT) (p=0.003) levels were higher in high BMI participants. BMI negatively correlated with FSH (p=0.009) and SHBG (p=0.001); and positively correlated with FT (p=0.001) and TT (p=0.002).

**Conclusion:**

Peculiarities of menstrual function and hormonal changes in young women with thinness or obesity since childhood are related to the types of reproductive disorders and their childhood BMI.

## Introduction

Body mass index (BMI) has been related to reproductive health (1). An extreme of body mass adversely affects reproductive function, starting from pubertal development, menstrual function and fertility. Body fat has a regulatory role in reproduction (2). Except for endocrine glands (adrenal cortex and gonads), sex steroids are produced by fat cells (3). Large amounts of fat cells produce respectively large amounts of estrogens (causes the organism to react as if in case of contraception). The lack of fat tissue causes lack of estrogens and thus disruption of menstrual cycle and ovulation (4, 5). Age of menarche varies internationally and especially in less developed countries. A cross well-nourished a population in developed countries, median age at menarche is between 12 and 13 years (6). 

The critical weight hypothesis states that the onset and regularity of menstrual function necessitates maintaining weight above a critical level, therefore above the critical amount of body fat (7). Higher gain in BMI during childhood is related to an earlier onset of puberty and menarche (8-10). So the age of menarche tends to be lower in obese and higher in underweight compared to girls with normal weight, which may have implications for subsequent reproductive problems. For example, Sundell *et al* reported that more severe dysmenorrhea was associated with earlier menarche (11). Rapid weight loss, especially due to stressful situations, eating disorders and excess physical activity, leads to hypothalamic amenorrhea (hypogonadotropic hypogonadism), ovulation problems, exhaustion of follicles and thus infertility (7, 12, 13). 

It is well known that obesity causes different health risks, including gynecological-endocrine problems such as hyperinsulinemia, insulin resistance, hyperandrogenism, anovulation, polycystic ovary syndrome (PCOS), and infertility and in case of achieving pregnancy- following obstetric risks (7). In case of pregnancy the risk of spontaneous abortion is increased both in pre-pregnant underweight and overweight or obese women (14, 15). Most of the studies regarding the association between BMI and reproductive health refer to rapid weight loss or rapid weight gain or that associated to eating disorders (16, 17). But there are not enough and consistent evidence proving whether elevated risks are related to being overweight or underweight in childhood. 

So it was interesting to investigate the relationship between childhood BMI with subsequent reproductive problems and respective hormonal changes in female patients with current BMI problems.

## Materials and methods

This cross-sectional prospective study was approved by the local ethical committee of the I. Zhordania Institute of Human Reproduction and informed consent was obtained from all participants. One hundred and three young women (sample size was calculated based on 95% confidence interval that reflects a significance level of 0.05) (12-30 years old) with BMI≥25 kg/m^2^ (overweight) or BMI˂18.5 kg/m^2^ (underweight) who reffered to our clinic -I. Zhordania Institute of Human Reproduction, with different reproductive problems (menstrual disorders, acne, excess body hair, etc.), between the periods of September 2012to April 2014, were recruited for the study. 

Our inclusion criteria were 12-30 years old women (at least 2 years past after menarche) BMI˂18.5 or ≥25 kg/m^2^ and childhood thinness or childhood obesity, that was obtained from the history records of their pediatricians. The following exclusion criteria were used: any type of chronic disease; eating disorders; hormonal therapy or contraception in the 6 months preceding the study. The participants who did not have full history records (particularly BMI record since childhood) were also excluded. 

All of the participants underwent clinical examination including measurement of height, weight and, BMI (weight divided by square of height- kg/m^2^). Age of body weight changes, menstrual disturbances, assessment of hirsutism (modified Ferriman and Gallwey score >8), stretch marks (white or colorful striae), pigmentation (acanthosis nigricans), acne (mild to severe), fertility problems were recorded. The sexual development was assessed by the Tanner scale. Gynecological ultrasound was held by Volusson S6 in all the participants. The diagnoses were established according to the respective clinical-hormonal-instrumental data. PCOS was diagnosed according to the Rotterdam 2003 criteria. 

Blood sampling was performed in the early follicular phase (between days 2 and 5 after the last menstrual period). The following hormonal analyses were performed in all subjects: follicle-stimulating hormone (FSH), luteinizing hormone (LH), estradiol (E_2_), total testosterone (TT), prolactin (PRL) (MiniVidas Analyzer, Biomerieux sa France, Italy), free testosterone (FT), sex hormone binding globulin (SHBG), dehydroepiandrosterone sulfate (DHEA-S), 17-hydroxyprogesterone (17α-OHP) (IFA, HumaReader HS, Germany). Cortisol (IFA, HumaReader HS, Germany) was measured in 57 patients. Insuline resistance index (HOMA-IR) was analyzed in overweight and obese patients. In 4 cases detection of karyotype was performed. 

**Statistical analysis**

Statistical analysis was held by IBM SPSS 20. A p<0.05 was considered significant, with confidence interval of 95%. Comparisons of two independent groups were made using the Student’s *t*-test, Chi-square or F-Test (analysis of variance ANOVA to analyse difference between group means. Correlation analysis was held by Pearson correlation test.

## Results

The mean age of participants did not differ between the groups of underweight (n=48, mean age 20.1 yr) and overweight (n=55, mean age 20.9) patients. Distribution of participants according to BMI in study groups is given in the table I. All of the underweight (n=48) or overweight and obese (n=55) women had BMI problems from childhood, but 63.6% of overweight and 33.3% of underweight patients mentioned deterioration of their respective BMI (losing or gaining more weight) since the period of menarche. The correlation was established between the onset of menstrual disruption and progression of changes in body mass (R=0.45, p=0.01). The mean BMI totaled 17.15 k/m^2^ (SD=0.91) (Range 15-18.49 k/m^2^) in the group of underweight patients and 31.30 k/m^2^ (SD=5.6) (Range 25-47 k/m^2^) in overweight patients.

The mean age of menarche totaled 12.95 y (SD=1.48) in the group of overweight and obese patients and 12.91 year (SD=1.63) in the group of underweight females, with no statistically significant difference (p=0.885). Regular menstrual cycle since the onset of menarche was detected in 5 (10.4%) underweight patients and 11 (20%) overweight patients. The rest of patients had different types of menstrual disorders, with no statistically significant distribution between the study groups (Table II). Hirsutism (p<0.05), stretch marks (p<0.001) and acanthosis nigricans (hyperpigmentation) (p<0.001) were exhibited significantly more frequently in the patients with high BMI, whilst distribution of acne was almost the same in the study groups (p=0.21). 

Only a small minority of patients were sexually active; 9 (18.7%) patients with low BMI and 19 (34.5%) patients with high BMI. Infertility was observed in 4 (8.4%) underweight and 16 (29.1%) overweight patients (p=0.03). Distribution of the patients according to the diagnoses is given in the table III. In the group of low BMI patients non-classical congenital adrenal hyperplasia (NCAH) and ovarian dysfunction turned out to be most frequent of all diagnosis, whilst in the group of high BMI patients PCOS and metabolic syndrome prevailed. 46 patients had PCOS, but metabolic disorders, insulin resistance (HOMA-IR >2.77) and obesity (BMI >30 k/m^2^) were particularly expressed in 25 patients. 

Comparison of hormonal parameters showed that the mean levels of FSH and SHBG were higher in the group of low BMI females than in the group of high BMI females; whilst in overweight patients FT and TT levels were two times as much as the same data in underweight patients (Table IV). According to the correlation analysis, it was established that BMI correlates with FSH, FT, TT, and SHBG. BMI negatively correlates with FSH (R=-0.30, p=0.01) and SHBG (R=-0.38, p=0.00) and positively correlates with FT (R=0.36, p˂0.001), TT (R=0.33, p=0˂0.001) (Table V).

**Table I:** Distribution of patients according to BMI in study groups (%)

**BMI k/m** ^2^	**Underweight n (%)**	**BMI k/m** ^2^	**Overweight n (%)**	**p-value**
<16	6 (12.5%)	25-29.9	27 (49.1%)	˂0.001 (#)
16-16.99	8 (16.7%)	30-34.9	15 (27.3%)	
17-18.49	34 (70.8%)	35-39.9	7 (12.7%)	
18.5-24.99	0	>40	6 (10.9%)	
Total n(%)	48 (100%)	Total n (%)	55 (100%)	

BMI Classification according to WHO 2004.

Chi-square test.

(#) P-value <0.05 considered significant .

**Table II:** Distribution of patients according to menstrual patterns in study groups

**Menstrual Function**	**BMI ˂18.5**	**BMI ≥25**	**p-value**
Regular Cycle	5 (10.42)	11 (20.00)	0.340
I Amenorrhea	6 (12.50)	2 (3.64)	
II Amenorrhea	7 (14.58)	8 (14.55)	
Oligomenorrhea	20 (41.67)	23 (41.82)	
Dysmenorrhea	3 (6.25)	4 (7.27)	
Menorrhagia	2 (4.17)	5 (9.09)	
Menometrorrhagia	5 (10.42)	2 (3.64)	
Total	48 (100)	55 (100)	

Data are presented as n (%).

Note: Chi-square test

P-value <0.05 considered significant

**Table III:** Distribution of patients according to the diagnosis in the study groups

**Diagnosis**	**BMI <18.5**	**BMI≥25**	**p-value**
PCOS	3 (6.25)	21 (38.18)	˂0.001 #
Metabolic Syndrome &PCOS	0 (0.0)	25 (45.45)	
NCAH	14 (29.17)	0 (0.0)	
Ovarian Dysfunction	10 (20.83)	2 (3.64)	
Hyperprolactinemia	6 (12.50)	5 (9.09)	
Dysmenorrhea	4 (8.33)	0 (0.0)	
Eugonadotropic Amenorrhea	5 (10.43)	1 (1.82)	
Premature Ovarian Failure	1 (2.08)	0 (0.0)	
Mullerian Agenesis	1 (2.08)	0 (0.0)	
Gonadal Dysgenesis	1 (2.08)	0 (0.0)	
Endometriosis	2 (4.17)	1 (1.82)	
Ovarian Follicular Cyst	1 (2.08)	0 (0.0)	
Total	48 (100)	55 (100)	

Data are presented as n (%).

Chi-square test.

PCOS: Polycystic ovarian syndrome

NCAH: Non-classical congenital adrenal hyperplasia

(#) P-value <0.05 considered significant.

**Table IV:** Hormonal patterns in study groups

**Hormonal Analyses (Normal range)**	**BMI <18.5**	**BMI≥25**	**p-value**
FSH (3-12 IU/I)	10.7 ± 6.1	7.89 ± 3.57	0.013 #
LH (0.8-10.5 IU/I)	9.76 ± 6.3	7.49 ± 5.2	0.099
E_2_ (13-191 ng/ml)	37.6 ± 23.1	40.3 ± 32.4	0.691
FT (0.5-1.7 ng/ml)	1.88 ± 0.98	2.59 ± 1.45	0.019 #
TT (0.1-0.9 ng/ml)	0.65 ± 0.5	1.07 ± 0.8	0.003 #
SHBG (15-120 nmol/l)	55.2 ± 40.4	25.6 ± 20.0	0.000 #
DHEA-S (0.8-3.9 U/ml)	3.45 ± 1.4	3.73 ± 1.17	0.414
17-OHP (0.3-1.0 ng/ml)	1.05 ± 0.56	1.05 ± 0.56	0.992
PRL (1.2-19.5 ng/ml)	19.3 ± 13.8	18.24 ± 10.6	0.678
Cortisol (50-250 pg/ml)	169.15 ± 27.08	213.58 ± 89.22	0.549

Note: Results are expressed as mean SD (standard deviation)

FSH: follicle-stimulating hormone

LH: luteinizing hormone

E_2_: estradiol

FT: free testosterone

SHBG: sex hormone binding globulin

TT: total testosterone

DHEA-S: dehydroepiandrosterone sulfate

17-OHP: 17-hydroxyprogesterone

PRL: prolactin.

F-test

FSH and other hormonal parameters of the patient with gonadal disgenesis was not included in statistical analysis.

(#) P-value <0.05 considered significant

**Table V:** BMI correlations with hormonal parameters

**Hormonal Parameters**	**R**	**p-value**
FSH	-0.300	0.009 #
LH	-0.207	0.078
E_2_	-0.141	0.229
DHEA-S	0.070	0.590
17-OHP	0.002	0.985
PRL	-0.089	0.415
FT	-0.364	0.001 #
TT	0.327	0.002 #
SHBG	-0.386	0.001 #
Cortisol	0.157	0.767

Note: Pearson correlation analysis

FSH: follicle-stimulating hormone

LH: luteinizing hormone

E_2_: estradiol

FT: free testosterone

SHBG: sex hormone binding globulin

TT: total testosterone

DHEA-S: dehydroepiandrosterone-sulfate

17-OHP: 17-hydroxyprogesterone

PRL: prolactin

(#) P-value <0.05 considered significant

## Discussion

Most evidence proving the adverse effects of extreme BMI refers to current BMI, rapid weight changes, or anything related to eating disorders and stressful situations such as excessive physical activities (7, 12, 13, 16, 17). In our study groups we included patients with childhood thinness or childhood obesity. We found very interesting results in terms of age of menarche, which was not different between the patients with childhood extreme BMI; that does not support existing evidence, proving that the onset of menarche takes place earlier in the overweight population and later in the underweight females compared to the normal weight population (8-10, 18). Evidence exists that when training starts before menarche, the latter can be delayed by as much as 3 years and the subsequent incidence of menstrual irregularity is higher (19).

It was quite interesting that we could not find a significant difference regarding the menstrual disorders in study groups, although the tendency of amenorrhea and menometrorrhagia was revealed in low BMI patients. Low weight and weight loss is known to be associated with ovulatory dysfunction and thus infertility. Even a moderate weight loss of 10-15% under ideal body weight can result in menstrual irregularity (20, 21). 

Rapid weight loss and undernourishment leads a woman’s body into a state of emergency. Excess training, undernourishment and low BMI adversely affects reproductive function and thus fertility (12). Exercise induced amenorrhea is mostly attributed to hypothalamic amenorrhea (13). Hypogonadotropic hypogonadism, which is characteristic of rapid weight loss associated with stressful situations, eating disorders or excessive training, was not detected in our patients who were underweight from childhood. It was very interesting that FSH negatively correlated with BMI according to our data. 

The correlation was established between the onset of menstrual disruption and progression of BMI changes, that partially supports the existing evidence, but it is worth mentioning that our patients were underweight or overweight from childhood and some of them just ‘deteriorated’ their BMI since/after menarche. However existing evidence mostly focuses on rapid excessive weight gain or weight loss. Diagnoses of investigated patients turned out to be consistent with typical BMI changes. It is known that PCOS is characteristic of patients with high BMI. In addition, there is evidence suggesting that childhood obesity impacts the development of adolescent PCOS. It is also known that NCAH is not connected to high BMI (22, 23). 

Analyses of hormonal parameters revealed an interesting fact: despite having no significant difference in E_2_ levels between overweight and underweight patients, BMI was negatively correlated with SHBG and positively correlated with testosterone. This fact can be explained by insulin resistance and hyperinsulinemia in overweight/obese patients with persistent anovulation and PCOS, which augments ovarian/adrenal androgen production and SHBG suppression, thereby increasing androgen bioavailability (7, 22). The fact that NCAH prevailed in underweight patients, while there was no significant difference in DHEA-S and 17-OHP levels between the groups can be explained by the prevalence of metabolic disorders and PCOS in overweight patients. Increases in DHEA-S and 17-OHP in the latter group might be from ovaries or as a result of activation of HPO axis (hypothalamus-pituitary-ovary axis) (7, 23, 24). 

So the results of our study shows that the characteristics of reproductive disorders, age of menarche, menstrual patterns, diagnoses and hormonal levels are different in patients who have childhood BMI problems, compared to those who demonstrate substantial weight changes associated with stress, eating disorders and exercise. It would be interesting to collect a broader range of data (recruit more patients), considering the onset and type of BMI changes, to assess the fertility and pregnancy outcome risks and work out special treatment protocols.

## Conclusion

In conclusion, according to the results of our study the age of menarche and different menstrual disorders do not significantly differ in underweight and overweight or obese patients, although the progression of BMI changes correlates with the onset of menstrual disruptions. So peculiarities of menstrual function and hormonal changes in young females with thinness or obesity since childhood are related to the types of reproductive disorders and their childhood BMI.

## Note

***This article extracted from M.D. thesis. (*** ***Nutsa Aladashvili-Chikvaidze*** ***)***
